# Transsynaptic modulation of cerebellar nuclear cells: theta AC-burst stimulation

**DOI:** 10.1088/1741-2552/ad9ad1

**Published:** 2024-12-13

**Authors:** Qi Kang, Amir Roshani Talesh, Eric J Lang, Mesut Sahin

**Affiliations:** 1 Biomedical Engineering Department, New Jersey Institute of Technology, Newark, NJ, United States of America; 2 Department of Neuroscience and Physiology, and Neuroscience Institute, New York University Grossman School of Medicine, New York, NY, United States of America

**Keywords:** transcranial AC stimulation (tACS), tDCS, AM-tACS, theta-gamma stimulation

## Abstract

*Objectives.* Transcranial alternating current stimulation (tACS) and its variants are being tested in clinical trials for treatment of neurological disorders, and cerebellar tACS (ctACS) in particular has garnered much interest because of the involvement of the cerebellum in these disorders. The main objective of this study was to investigate the frequency tuning curves for the entrainment of the Purkinje cells (PCs) and the cerebellar nuclear (CN) cells by their axonal projections. In addition, we aimed to investigate the temporal and steady-state characteristics of the PC-CN transsynaptic modulation under clinically relevant stimulation waveforms. *Approach.* Experiments were conducted in anesthetized rats with the electrical stimulations applied to the cerebellar cortex while the spiking activity of PC and CN cells were recorded extracellularly. The PC-CN modulation was tested in a wide range of AC frequencies (1–1000 Hz). Furthermore, high-frequency AC stimulation (40–400 Hz) repeated at 4 Hz, that we termed *theta AC-Burst Stimulation*, was tested for its transient and steady-state responses. *Main results*. The CN cell firing patterns suggest that the population of projecting PCs that is entrained by the surface stimulation consists of the cells that are entrained in 180° opposite phases to each other. The CN cell spiking activity in general follows the entrainment pattern of the projecting PCs in the transient response. The CN entrainment during the steady-state turns into suppression at high frequencies of the stimulation. The PC responses could be explained with a simple statistical model that suggested that low-frequency (as well as DC) and high-frequency AC modulation may be operating through different neural mechanisms. *Significance.* High-frequency AC stimulation with a low-frequency envelope can be leveraged to induce CN modulation at theta frequencies. These results may explain some of the clinical findings and provide insight for future clinical trials of ctACS.

## 1. Introduction

The cerebellum is a novel brain site for transcranial alternating current stimulation (tACS) applications. The cerebellum is not only involved in motor but also cognitive functions and emotional behavior [1–4] with its projections to the cerebral areas through the thalamus. Thus, cerebellar tACS (ctACS) may become a treatment option for a plethora of motor and non-motor neurological disorders in which the cerebellum is implicated.

The earliest report on ctACS was only a decade ago demonstrating that tremor frequencies could be entrained in healthy subjects [5]. More recently, the effects of different AC frequencies were investigated on the excitability of the contralateral motor cortex via cerebellum-brain inhibition (CBI) [6, 7], where 50 Hz and 300 Hz ctACS increased excitability, but a low-frequency (10 Hz) ctACS did not. In agreement with these reports, 50 Hz ctACS improved motor skills [8], and 70 Hz ctACS improved visuomotor performance, but 20 Hz stimulation did not [9]. However, findings contradicting these results have also been reported. Two different studies found that ctACS could effectively strengthen CBI, i.e. induce a greater inhibition of the motor cortex [10, 11], at 5 Hz, but not at 50 Hz [10]. In order to explain the contradictory results in these clinical studies, it is crucial to understand the temporal and frequency-specific effects of ctACS on the Purkinje cells (PCs) first, which are the cells primarily affected by the transcranial application of the AC stimulation, and then demonstrate how modulation of PC simple spike (PC-SS) activity can alter the cerebellar nuclear (CN) cell spiking activity, which is the sole output of the cerebellum to the cerebral areas in the brain.

The reports of ctACS on the neural activity at the single cell level are scarce. We have reported that PCs of the cerebellar cortex could be entrained to the main frequency or the subharmonics of the AC stimulation applied to the rat cerebellar cortex [12]. We have also shown that a CN cell can be entrained transsynaptically via PC modulation [13]. Interestingly, the CN entrainment had a tuning curve where the AC stimulation in middle frequencies (50–250 Hz) was more effective. This raises the question whether the CN tuning curve is an emergent characteristic of the CN cell properties or that of the projecting PCs. Therefore, one objective of this study was to investigate the PC and CN entrainment profiles in a comparative manner in a wide range of stimulation frequencies. As a corollary to this, we also investigated the temporal characteristics of the PC-CN transsynaptic modulation under current pulses, and more importantly for some of the stimulation waveforms that are clinically relevant, where several cycles of high frequency sinusoidal waveforms are turned on and off intermittently at theta frequencies. We prefer to call this stimulation pattern *theta AC-Burst Stimulation* (TACBS) to distinguish it from theta-gamma stimulation [14–16] where a gamma frequency sinusoidal waveform is superpositioned on either or both half cycles of another low frequency sinusoidal waveform. The results have interesting implications for the choice of the stimulation waveforms for effective modulation of the cerebellar nuclei.

## 2. Methods

### 2.1. Animal preparation

Twenty three Sprague Dawley rats (200–350 g, 2 females, Charles River) were used in this study. All experiments were approved and performed in accordance with the National Institute of Health Guide for the Care and Use of Laboratory Animals. All procedures were approved and performed in accordance to the guidelines of the Institutional Animal Care and Use Committee, Rutgers University, Newark, NJ. Animals were initially anesthetized with 5% isoflurane in an induction chamber, and then moved to stereotaxic frame. The animal was switched to ketamine/xylazine mixture (80 mg kg^−1^ and 8 mg kg^−1^, IP) during the course of surgery and data collection. Additional doses of ketamine (15 mg kg^−1^, IP) were injected as needed based on the pedal reflexes. Body temperature was measured by a rectal probe and regulated with a heating pad under the animal (ATC 1000, WPI, Sarasota, FL). The blood oxygen level, monitored with a pulse oximeter attached to the hind paw, was kept above 90% during the data collection. The hair over the head was shaved and a midline skin incision was made to expose the skull over the cerebellum. The entire posterior side of the cerebellum was opened with rongeurs. The dura mater was left intact and covered with a silicon sheet (125 *μ*m) to prevent dehydration and cooling of the cerebellar cortex. Neural recordings were made inside a Faraday cage to eliminate electromagnetic interference.

### 2.2. Electrical stimulation of the cerebellar cortex

**Figure 1.**
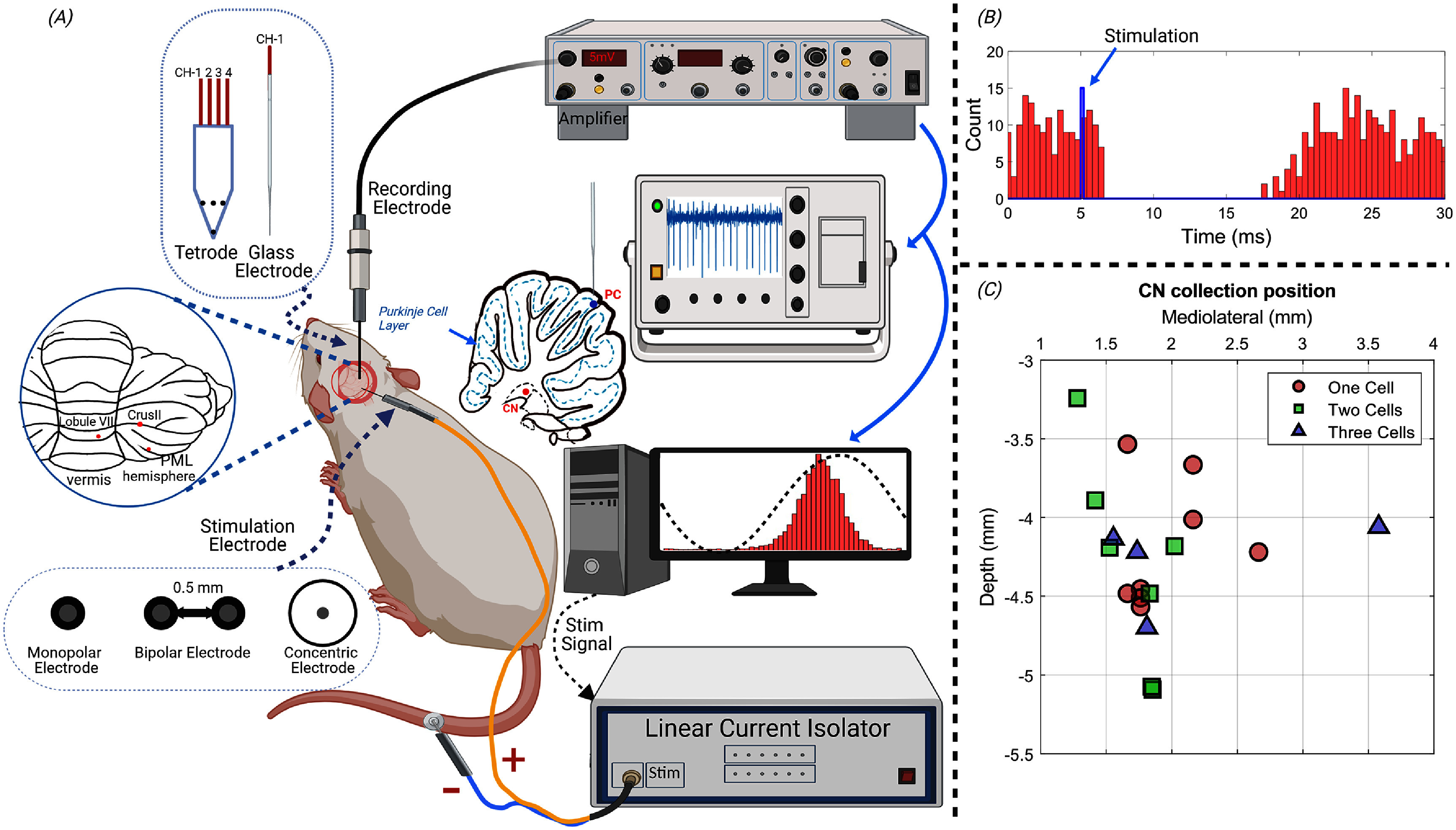
(A): Experiment setup for stimulation of the cerebellar cortex and recording from both PCs and CN cells. The posterior view of cerebellum depicts where the cortex was stimulated at crus II, PML and the vermis. Three different types of stimulation electrodes are shown with cross section images (monopolar, bipolar, and concentric electrodes). The recording positions in the cortex (blue dot) and the CN (red dots) are marked in the parasagittal drawing of the cerebellum. (B): Synaptic connectivity from the stimulated cortical area to the recorded CN cell was verified with a 10 Hz current pulse applied through the same cortical electrode used for AC modulation. The stimulation pulse (blue), and spike histogram triggered by the stimulus (red bars) are shown. The CN cell spikes are suppressed with a 2–3 ms delay that can account for the propagation time from the cortex to the CN. (C): The estimated position of all 34 CN cells, based on the stereotaxic coordinates in the coronal plane at AP = −2.5 mm form the interaural line. Multiple cells at a location indicates that the cells are isolated by clustering from the same recording.

Stimulus waveforms were generated in MATLAB (MathWorks, MA), sent out through a data acquisition card (PCI-6255, National Instr., TX), and passed through a V/I converter (Caputron, Hillsborough, NJ) before being applied to the cerebellar surface via a stimulus electrode (figure 1(A)).

Monopolar electrodes were used for stimulation unless indicated otherwise. The monopolar stimulation electrode was a silver wire (0.25 mm diameter) with the tip sanded with 220 grit paper to obtain a flat circular footprint, and coated with AgCl by immersing it in 1.84% sodium hypochlorite solution overnight. A thin plastic sheet encircled the wire to ensure that the current enters the cortex only through the tip. The circular tip was pressed against the dura to deliver the electrical stimulation at different points across the posterior cerebellar cortex (figure 1(A), inset). A disposable electromyogram electrode was attached to the tail as the return electrode for the stimulus current. The 1 kHz AC impedance between the two electrodes was typically around 12 kΩ. For bipolar stimulation, two similar monopolar electrodes were used with a mediolateral separation of 0.5 mm, edge to edge (figure 1(A)). In some rare cases, the stimulation was done by concentric electrodes, which were prepared by inserting a silver wire (0.125 mm diameter) into an 18-gauge needle. The space between the silver wire and the needle wall was filled with plastic material and the electrode tip was cut flat and sand papered before coating with AgCl (figure 1(A)). The central silver wire worked as a stimulus electrode and the outer layer (needle wall) as the return electrode. Monopolar electrodes were used in 23 cells (CN = 9 and PC = 14), bipolar electrodes in 34 cells (CN = 23 and PC = 11), and concentric electrodes in 8 cells (CN = 2 and PC = 6). Bipolar and concentric designs would be preferable over the monopolar configuration in order to contain the stimulation current more locally in the cortex. Nonetheless, the results with all three types of electrodes were lumped together because no difference was seen in the modulation characteristics.

### 2.3. Electrophysiological data collection

A tetrode (1–2 MΩ, AN00021, Thomas Recording Scientific Resources, Germany) was used for recording units in the CN and a glass micropipette electrode (3–5 MΩ) filled with normal saline was used for PC recording in most cases. For CN recording, the electrode was inserted through a cranial hole above the cerebellum at a lateral angle of 20° with the vertical axis and a rostrocaudal position of −2.5 mm from the interaural line to target the interpositus nucleus. The estimated mediolateral positions and the depths of the recorded cells are shown in figure 1(C). A total of 34 CN cells from 12 rats and 31 PCs from 12 rats (1 overlap with CN rats) were recorded.

Neural signals were amplified (Model 1700, A-M System, Carlsborg, WA) by a gain of 100 or 1000, and sampled at 100 kHz onto the computer via a data acquisition board (PCI-6255, National Instr.). Neural signals were monitored simultaneously on an oscilloscope and an audio speaker. The trials consisted of 40–140 s of stimulation at frequencies ranging from 1 Hz to 1000 Hz and amplitudes from 10 *µ*A to 50 *µ*A, unless specified otherwise.

Before collecting CN data, anodic and cathodic monophasic current pulses (10 Hz, 200–300 *μ*A, pulse width 200 *μ*s) were applied on the cerebellar cortex to ensure that the PCs in the vicinity of the stimulation electrodes were projecting to the CN cell that was recorded (*shock test,* figure 1(B)). The paravermal area in crus II (∼2 mm from the paravermal vein), and in rare cases the paramedian lobule (PML) and lobule VII of the vermis (figure 1(A), inset) were found to produce the strongest CN inhibition with the shock test and the lowest thresholds for CN modulation with AC stimulation. The connectivity was confirmed in each of the CN cells with a spike-triggered histogram that showed a period of silence or substantially decreased spiking probability following the stimulus pulse (figure 1(B)).

### 2.4. Data analysis

**Figure 2.**
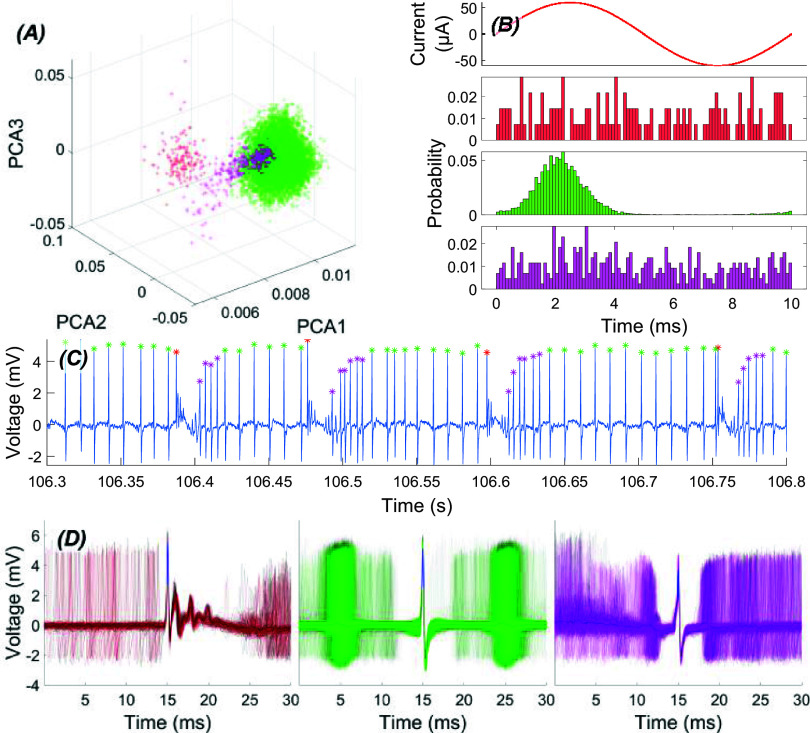
Clustering of spiking activity for a typical PC. (A): Scatter plots of the first three principal components of the spikes. CS: red, SS: green, SS that follow the CS: magenta. (B): Time locked probability distribution of PC spikes over the AC stimulation cycle for three different clusters. Only SS (green) are modulated. (C): PC firing pattern with CS and SS peaks are marked with color-coded dots. (D): Aggregate of signal traces triggered by each of the three different spike types, plotted with the same color codes.

All data processing was performed in MATLAB. Raw signals were band-pass filtered (100–10 kHz). Initially, stimulation artifacts in the signal were removed by taking the stimulus-triggered average of the raw signal in a moving window with a length of 40 cycles of the stimulation to extract the artifact waveform, and subtracting it from the raw signal in each cycle within the window. PC spikes were clustered (figure 2(A)) by manually drawing polygons on the scatter plots of the first two, the 1st and the 3rd, or in rare cases based on the first four principal components of single channel recordings made with glass electrodes. The CN spikes were clustered using an automated clustering algorithm (Gaussian mixture model in MATLAB) based on the first principal components from each one of the four tetrode channels. The action potential waveforms within 1.5 ms around its negative peak were taken for the principal component analysis.

Then, the time points of spikes from the selected cluster were plotted with respect to the AC stimulus phase as a histogram (figure 2(B)). The phase lock value (PLV) was defined [17] as PLV *=* abs(mean(e^i*θ*^)), where the *θ* are the phase angles of individual spikes with respect to the stimulation cycle, and used as an index of modulation or entrainment. The PLV is artificially reduced by cancellations of probabilities when the spiking activity peaks at two different phases of the cycle that are 180° apart (bimodal histogram). To classify the cells as bimodal or unimodal, we calculated the PLV at the stimulation frequency and at twice the stimulation frequency (PLV2), which computed the PLV in half cycles of AC, to avoid the cancellations. If the PLV2 was greater than PLV at 100 Hz stimulation frequency, a frequency that typically maximized the modulation, we categorized the cell as bimodal and used PLV2 instead of PLV for these cells.

**Figure 3.**
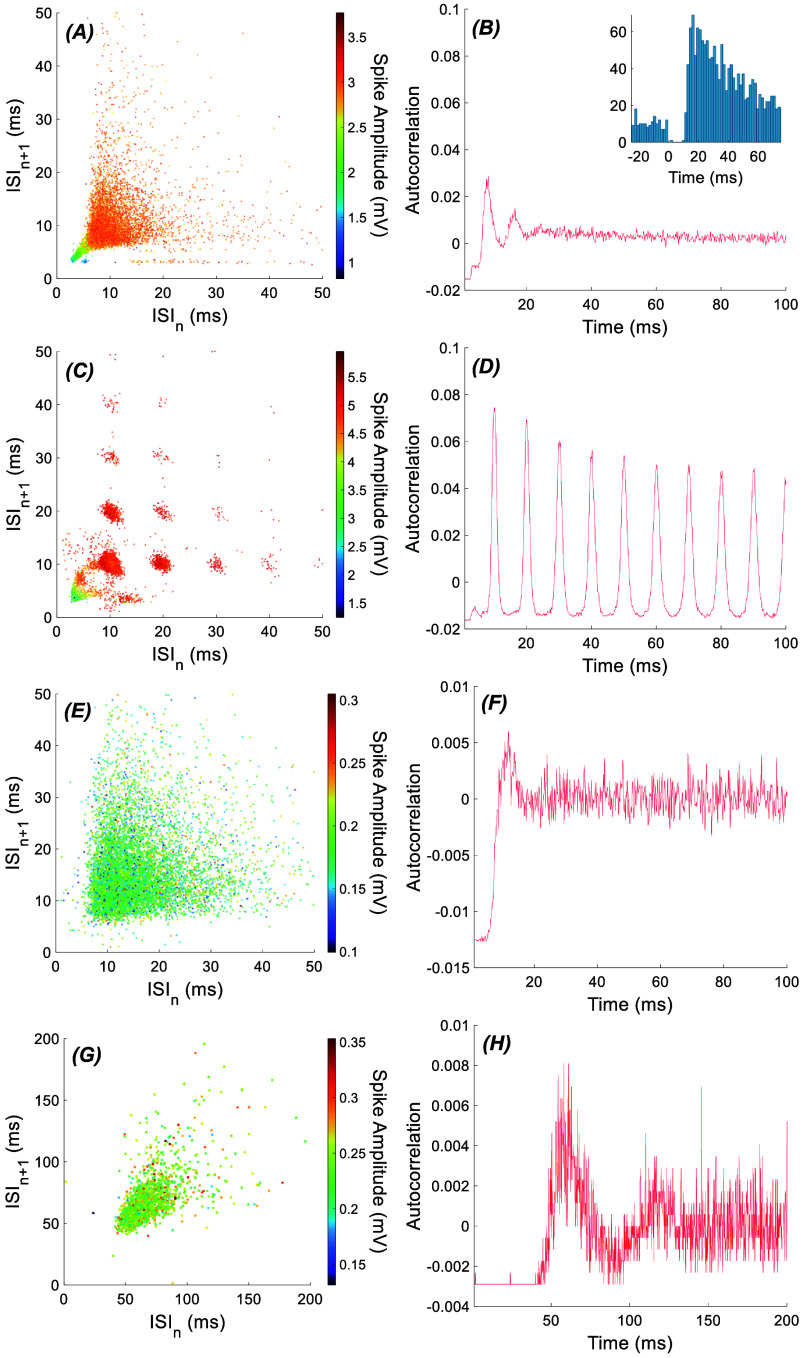
Typical firing patterns for a PC, a CN cell and an MLI. (A) & (B): ISI return map and autocorrelation for a typical PC (same PC as in figure 2). The histogram in the inset in B shows the characteristic silence in SS after each CS. (C) & (D): ISI return map and autocorrelation for AC stimulation of the same PC in A (E) & (F): ISI return map and autocorrelation for a typical CN, and (G) & (H): an MLI. In all autocorrelation plots, the time points of spikes are down sampled to 5 kHz resolution.

Inter-spike interval (ISI) plots and the spike amplitude histograms were used as criteria to ensure that the selected cluster contains spikes only from one cell. The ISI*_n_*−ISI*_n_*_+1_ plot (return plot, n is spike index) was utilized as an additional criterion, which produces a *V*-shape distribution for a single cell (figure 3). Spikes outside the *V*-shape distribution suggest the presence of another cell or background noise within the selection. Additionally, the spike amplitude histogram would typically follow a gaussian distribution. If not, the selection was decided to be contaminated with either another cell or background neural activity. By tracking the changes in the scatter plots from trial to trial, the user could determine if the selected cluster belonged to the same cell. To identify the cell as a PC, the presence of silence after complex spikes (CS) was used as the main criterion, along with the return plots (figure 3). If the cell was decided not to be a PC in recordings from the cortex, it was assumed to be a molecular layer interneuron (MLI).

## 3. Results

### 3.1. PC modulation by AC fields and pulses

Purkinje spiking activity can be separated into three categories based on the shape of the action potential and the ability of AC stimulation to entrain them (figure 2): CSs (red), SS (green) and SS that follow the CS after the silence (magenta). It is clear from the phase-locked probability distribution of spikes in these three clusters that only the SS are modulated by AC stimulation, and not the CS or the SS that immediately follow the post-CS silence. The CS, the post-CS silence, and the SS that come after show a pattern that is not affected by the AC stimulation. There is a smooth transition from a high-frequency, low-amplitude spikes to a baseline pattern after the silence period that is highly stereotypical and independent of the AC stimulation. This topic was spared for future investigation, and because the CS and the SS in the post-CS silence periods were a small fraction of the total number of spikes, an effort was not made to exclude them in the forthcoming analysis.

Plotting ISI*_n_*_+1_ against ISI*_n_* (return map, n is the spike number) produced a pattern that was unique to PCs where the high frequency SSs that occur after the CSs clustered at the bottom left of the plot, and they always had lower amplitudes than other SSs in our data (green dots in figure 3(A)). This CS-SS pattern is a characteristic of PCs, and not seen with CN (figure 3(e)) or MLI (figure 3(g)). The SSs cease to fire typically for 10 ms or longer after each CS in the PCs (figure 3(B), inset). With AC stimulation, the SS pattern changes where the ISIs concentrate around the points that correspond to the cycle length of the AC stimulation and its multiples (figure 3(C)). The SS auto-correlogram contrasted with the spontaneous one (figure 3(B)) also shows entrainment at the cycle length of the fundamental frequency (10 ms, 100 Hz) and its multiples (subharmonics, (figure 3(d)).

#### 3.1.1. PC entrainment vs. AC frequency

**Figure 4.**
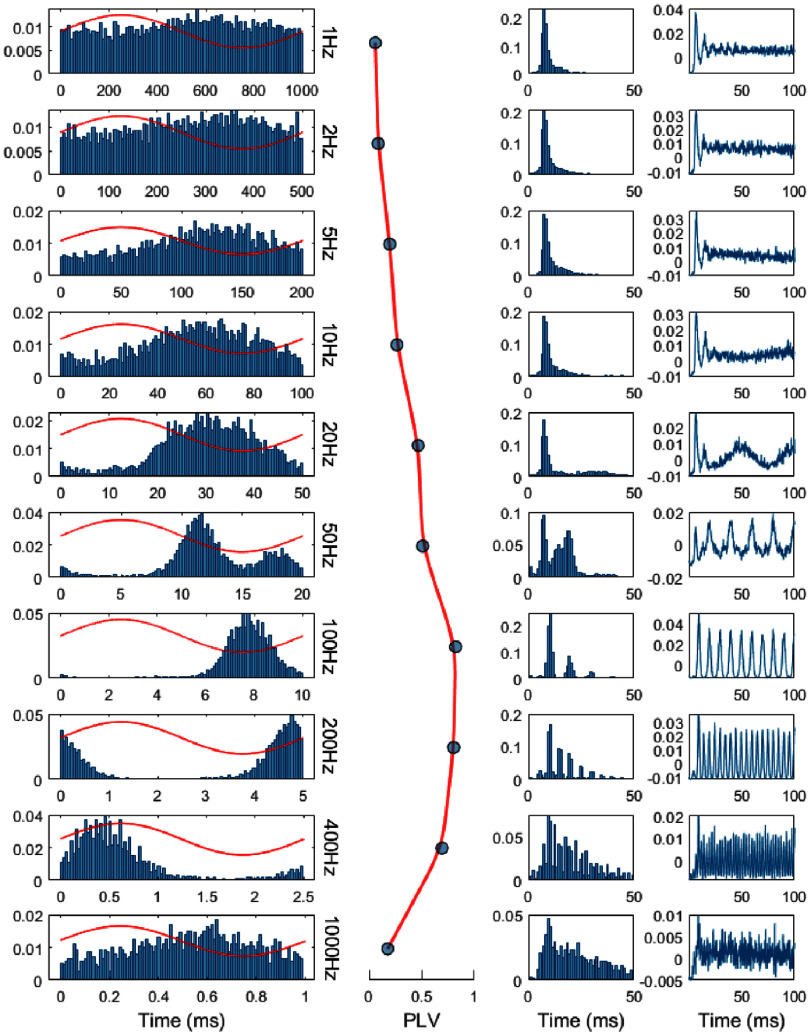
Response of a sample PC to AC frequencies from 1 Hz to 1000 Hz at 11 µA with a monopolar electrode. First column from Left: probability distribution of PC-SS triggered by the AC stimulation cycle. Red traces are the stimulus current waveform as it is applied to the cerebellum. Second column: corresponding PLVs at each frequency. Third column: ISI histogram of the SSs. Right column: autocorrelation plots, down sampled to 5 kHz.

Figure 4 shows the typical response of a PC to subthreshold AC stimulation where the modulation index (PLV) varies as a function of frequency and typically has a maximum in the mid-frequencies (here ∼100 Hz). In this cell, at stimulation frequencies below 50 Hz, the PC can fire multiple times per AC cycle, and the ISI plots reflect the spontaneous variations in the firing rates with a single peak at ∼8 ms. At 50 Hz, there are multiple peaks in the autocorrelation plot (right column) with separations that correspond to the AC cycle length (20 ms), indicating that there was one spike per cycle in a great majority of cycles. The cell begins to skip AC cycles as the stimulation frequency approaches and exceeds the spontaneous firing rate (∼125 Hz in this case), as it is evident from the peaks in the ISI plot at multiples of the AC cycle length (e.g. at 20 ms, 30 ms, and 40 ms for 100 Hz, etc). By skipping cycles, PC-SSs can entrain even at high frequencies of AC stimulation while keeping the same average firing rate. The timing of the spikes can be entrained more precisely as the frequency is increasing and the AC cycles are becoming shorter. This may consequently increase the synchrony amongst all the PCs entrained by the same stimulation. However, each PC may choose to fire in a different cycle of the AC stimulation (see discussion section on ‘spikes locking to subharmonic’). Entrainment starts to decrease again (PLV) at the higher end of the spectrum (400–1000 Hz), potentially because of the jitter in ISI due to parallel fiber inputs making it harder to entrain the PCs at millisecond resolutions. Another potential mechanism is that the time constant of the cell membrane may filter down the voltage oscillations induced by the stimulation at such high frequencies.

**Figure 5.**
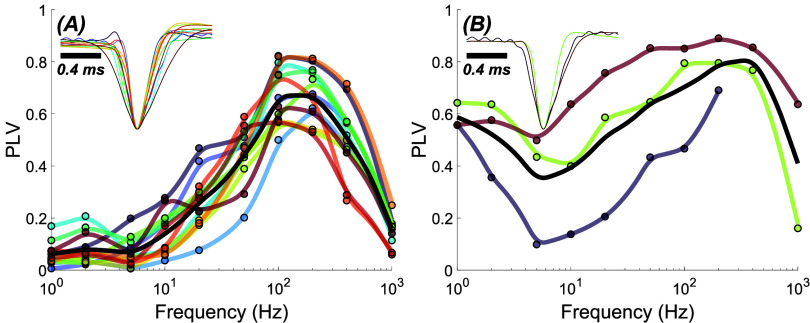
PLV-frequency plots of 15 PCs (color coded) from 6 different animals. Black lines are the polynomial fits to the average data. Action potential shapes for each cell are shown in the inset, which show no discernible difference that could help categorize these cells. A: PCs with poor modulation below 5 Hz (12 cells from 5 rats) and (B) those that have increasing PLV below 5 Hz (3 cells from 2 rats).

The PLV vs. frequency plot follows the same pattern in most PCs (figure 5(A)) except in a small number of cases (3/15, 20%) where the cell also modulates at low frequencies (figure 5(B)). In both cases, the PLV peaks and begins to fall around 100–200 Hz.

#### 3.1.2. Duality in the phase of the PC response

**Figure 6.**
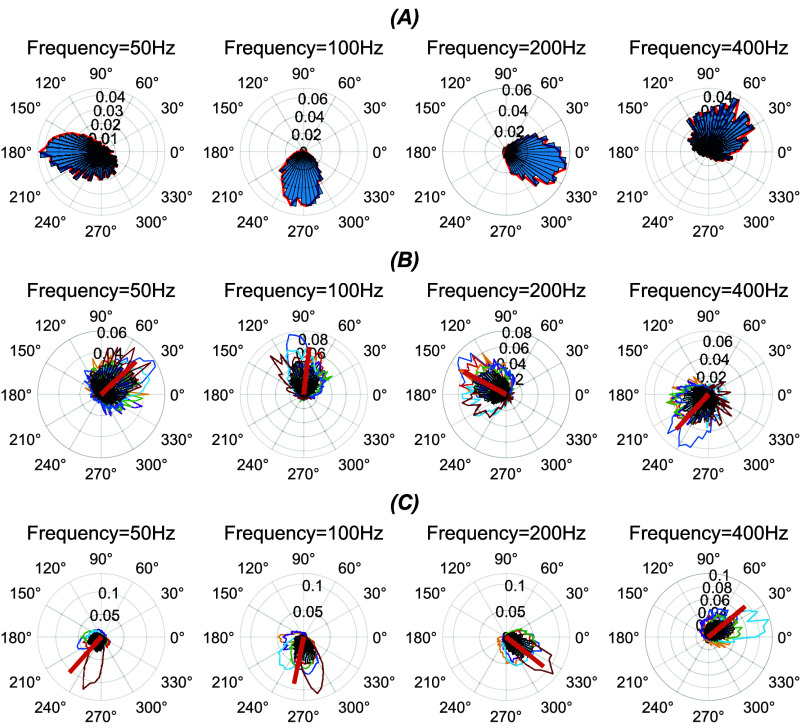
PC histograms in polar coordinates for 50, 100, 200, 400 Hz stimulation. (A): A sample PC where red contours show the envelopes of the histograms. (B): Aggregates of all the histogram envelopes for PCs with ∼0° phase shift (8 cells from 5 rats) and with (C) ∼180° phase shift (7 cells from 4 rats). The brown bars show the mean phase angle of all the spikes from all the cells.

PC histograms show two distinct behaviors where the entrainment to electrical stimulation in these two categories have a 180-phase shift with respect to each other in the AC cycle. Figures 6(B) and (C) show aggregates of these two types of responses from a total of 15 cells in 6 rats. The phase shift calculations are less accurate below 50 Hz, because the entrainment is weak (not shown). At high frequencies on the other hand the biological jitter in spike times become comparable to the AC cycle and appears as large variations in the phase. The mean phase angles from all cells (brown bars in figures 6(B) and (c)) have approximately 180° phase shift between the two types of PC at all frequencies. This observation suggests that PCs may modulate in opposite directions even when they are located near each other in the cerebellar cortex (see discussion).

#### 3.1.3. PC response to rectangular pulse stimulation: upward and downward PCs

**Figure 7.**
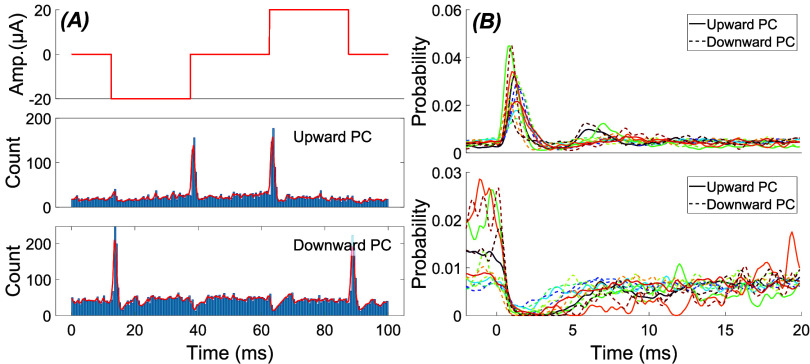
(A): Recording from two PCs showing two different modes of behavior (upward and downward cells) in response to 25 ms-long pulse stimulations. Top: stimulus current waveform. Middle and bottom: histograms for an upward and a downward PC. Red lines are the envelopes. (B): Aggregates of histogram envelopes from 13 cells in 9 animals. Top: excitatory responses and, bottom: inhibitory responses, to a transition that may be a rising (5 upward PCs) or falling (8 downward PCs) edge of the stimulus. Same color indicates the same cell in both figures in B.

From a system analysis perspective, a continuous sinusoidal input characterizes the system only for the steady-state response at a specific frequency. A rectangular pulse, on the other hand, can reveal the transient response of the system for sudden changes of the input. Note that there will also be a transient component in the response at the onset and offset of sinusoidal inputs as they are turned on and off. When we applied rectangular pulses to the cerebellar cortex, the PC firing rates showed strong transient changes at the rising and falling edges of the stimulus waveform. Interestingly, some PCs increased their firing rate transiently at the rising edge and decreased at the falling edge while the others had the opposite phase response, as exemplified in figure 7 with 13 cells of either type. The direction of the transient response matched the direction of modulation with AC stimulation in each cell. It is also interesting to note that the transient responses were very strong while the change in the mean firing rate during the flat portion of the stimulation was almost absent or very small. The flat portion of the rectangular pulse can be interpreted as the steady-state response of the cell to a DC stimulation if it is sufficiently long to settle to a baseline after the transient part is over. The DC response was consistently weaker than the transient response through all PCs in this study. The cells with the ability to modulate below 5 Hz (figure 5(B)) had a slightly higher firing rates in the flat portion of the rectangular pulse than the baseline, but still much lower than the transient response. Also, the transient response was primarily determined by the direction of the change alone in the stimulus waveform regardless of the DC offset. That is, a positive-going edge from negative to zero produced a similar response to the edge that goes from zero to positive (compare the two peaks in each cell, figure 7). From now on we will call the PCs that show excitatory response by the rising phase of the stimulation the *upward* cells and the PCs with the opposite response the *downward* cells. The transient increase or decrease in firing probability peaked within ∼1 ms of the pulse transitions, lasted about 2 ms, and settled to a baseline level within less than 10 ms (figure 7(B)).

### 3.2. CN cell modulation by AC fields and pulses

#### 3.2.1. CN cells have unimodal and bimodal histograms for AC stimulation

**Figure 8.**
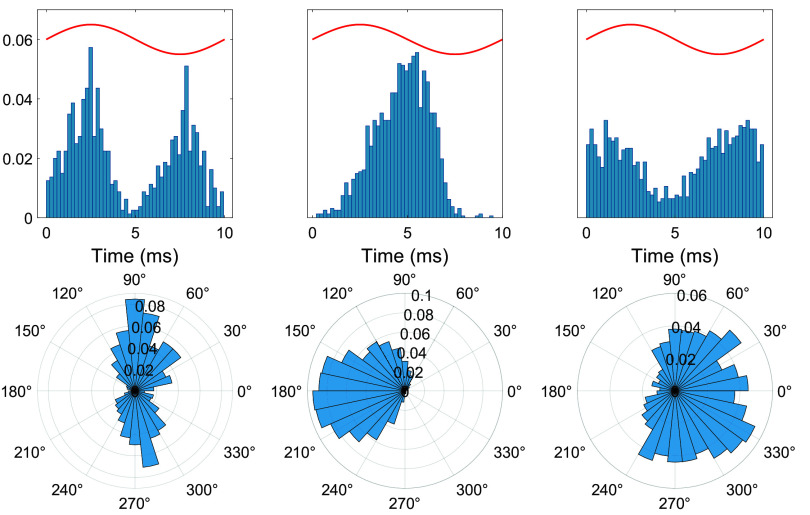
AC cycle triggered spike probability histograms for three different CN cells from three different rats showing the modality in response to 100 Hz AC stimulation. Left: bimodal, middle: unimodal ∼180° phase shift, Right: unimodal ∼0° phase shift.

CN cell spike probability histograms to sinusoidal stimulation had either bimodal (figure 8, left panel) or unimodal distributions. Interestingly, in unimodal responses there were two different types with 180° phase shift with respect to each other (figure 8, middle and right panels), similar to the case observed with the PC modulation. The bimodal case on the left is almost perfectly symmetrical, although in other cases one of the bumps was usually bigger than the other.

The bimodal CN histogram can be explained by the upward and downward PC responses. In fact, when we merged the histograms from all the PCs in figure 6 with two different responses, we could see a bimodal response like that of the bimodal CN cells (supplementary figure 1). This suggested that the bimodal CN cells were receiving inputs from both upward and downward PCs while unimodal CN cells were projected primarily by only one type of PCs. In the unimodal CN response, depending on which type of projecting PCs are dominating, we would have either upward unimodal or downward unimodal CN response. If there are similar number of upward or downward PC projections to the CN cell, the histogram should be bimodal with two similar size bumps that are separated by 180°. In some cases, one of the bumps was larger than the other, which suggested that the population of either the upward or downward PCs was larger than the other. Thus, modality was a spectrum varying from pure unimodal to symmetrical bimodal, and most cells were somewhere in between. Out of 26 CN cells, 11 were bimodal and 15 were unimodal. In the bimodal cells, the number of spikes in two opposite phases had ratios ranging from 1:4.7 (the most unbalanced case) to 1:1 (the most balanced case), with a mean of 1.14 ± 0.36 (*n* = 11). It should be mentioned here that a small component of the modulation may also be due to the direct effects of the e-field on the CN cell (see discussion).

#### 3.2.2. The CN responses to pulse stimulation is also unimodal or bimodal

**Figure 9.**
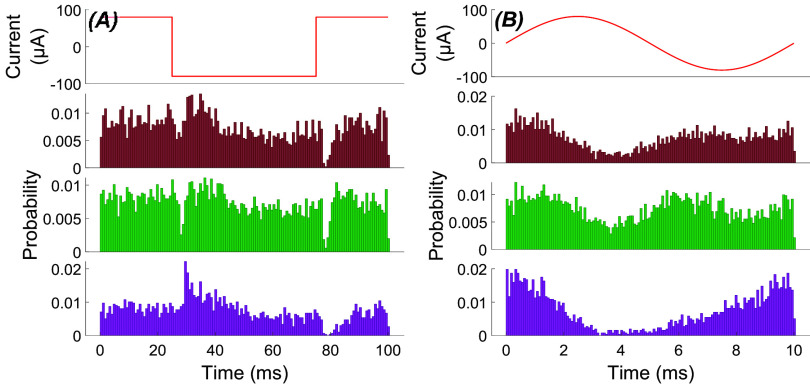
(A): Response of three different CN cells isolated from a single recording to 10 Hz biphasic pulse stimulation. (B): Response of the same cells to 100 Hz AC stimulation.

The CN responses to pulse stimulation can also be explained by upward and downward PCs. For example, figure 9(A) shows the response of three different CN cells, simultaneously recorded through the same tetrode, to pulse stimulation. The first two cells show inhibition at both rising and falling edges unlike the PC responses, which are always in opposite directions (excitatory and inhibitory or vice versa), at the rising and falling edges of the rectangular pulse stimulation. This may occur if the CN cell is projected by both upward and downward PCs; Because excitation is usually stronger in the PCs and they are inhibitory to the CN cells, the net response emerges as inhibition at both rising and falling edges of the stimulus. The inhibition at the rising edge is stronger than the falling edge in the first two cells, which must be because the number of upward PCs is larger than the downward ones that are projecting to these CN cells.

In contrast, the third cell shows excitation at the falling edge and inhibition at the rising edge, suggesting that this cell is projected by mostly the upward PCs, which are excited by rising edge of the pulse stimulation (hence inhibition of CN), and inhibited by the falling edge (hence excitation of CN). The response of the same CN cells to AC stimulation reveals that the first two cells have an asymmetrical bimodal response while the third cell is unimodal (figure 9(B)). Going forward, we will call the unimodal responses in which the CN is putatively projected by upward PCs, *upward unimodal*, and the ones that are putatively projected by downward PCs, *downward unimodal*.

### 3.3. Transient and sustained responses of CN cells

#### 3.3.1. CN response to TACBS has transient phases

**Figure 10.**
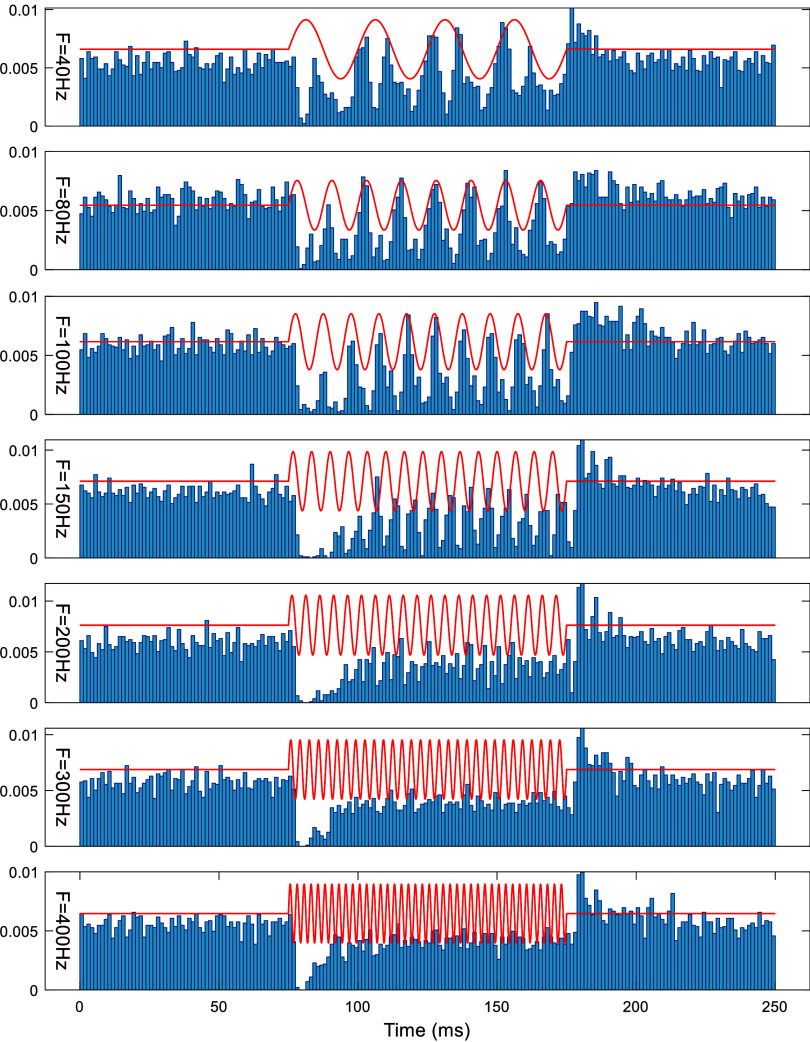
Response of a sample CN to theta AC-burst stimulation at 30 *µ*A where the theta frequency is 4 Hz, and the burst frequency is varied from 40 Hz to 400 Hz. Time locked probability distribution (sum of probabilities is unity, bin size = 1.25 ms) of CN spikes are plotted over the theta stimulation cycle. The AC-burst window is 100 ms for all frequencies. Red traces represent the stimulus current with a zero baseline.

Turning a sinusoidal waveform on and off can lead to transient effects at the start and the end of each block, despite the fact that the waveform has a zero mean. Indeed, there is an initial inhibition at the start of the AC-burst window, and there is either entrainment or suppression during the rest of the window depending on the burst frequency, and finally a rebound of activity after the burst window. In the sample cell shown in figure 10, the entrainment during the burst window is observed up to 200 Hz, although it gets weaker with frequency. Moreover, the average firing rate is reduced (suppression) with increasing frequency during the window. The duration of initial inhibition is longer at middle frequencies.

#### 3.3.2. Multiple phases of CN response to TACBS

**Figure 11.**
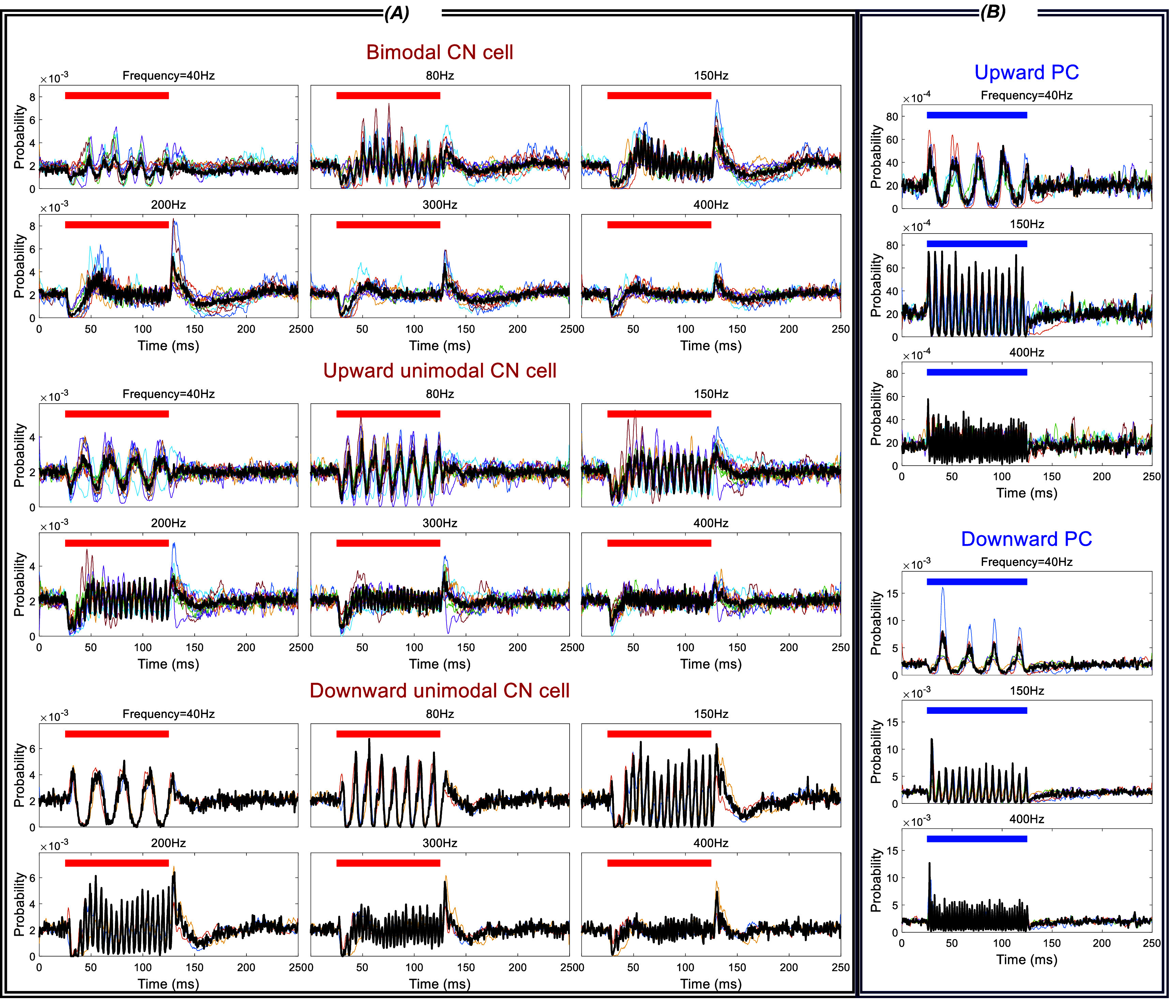
Aggregate of histogram envelopes from all CN and PC responses to theta AC-burst stimulation. (A): CN response to theta AC-burst stimulation with 4 Hz theta cycle and 6 different burst frequencies of 40, 80, 150, 200, 300, 400 Hz (26 cells from 9 rats). Top: bimodal (11 cells from 5 rats). Middle: upward unimodal (12 cells from 5 rats). Bottom: downward unimodal (3 cells from 3 rats). (B): PC responses to theta AC-burst stimulation with 4 Hz theta cycle and 3 different burst frequencies of 40, 150, 400 Hz (13 cells from 6 rats). Top: upward PCs (8 cells from 6 rats). Bottom: downward PCs (5 cells from 4 rats).

Figure 11(A) shows the aggregate of the CN response envelopes to TACBS from all the cells, separated into three categories: Bimodal, upward unimodal and downward unimodal. As we compare these results to TACBS responses of the PCs (figure 11(B)), we can begin to see how the upward and downward PC projections to the CN cells may give rise to these three different categories of the CN responses.

##### 3.3.2.1. Initial Inhibition

**Figure 12.**
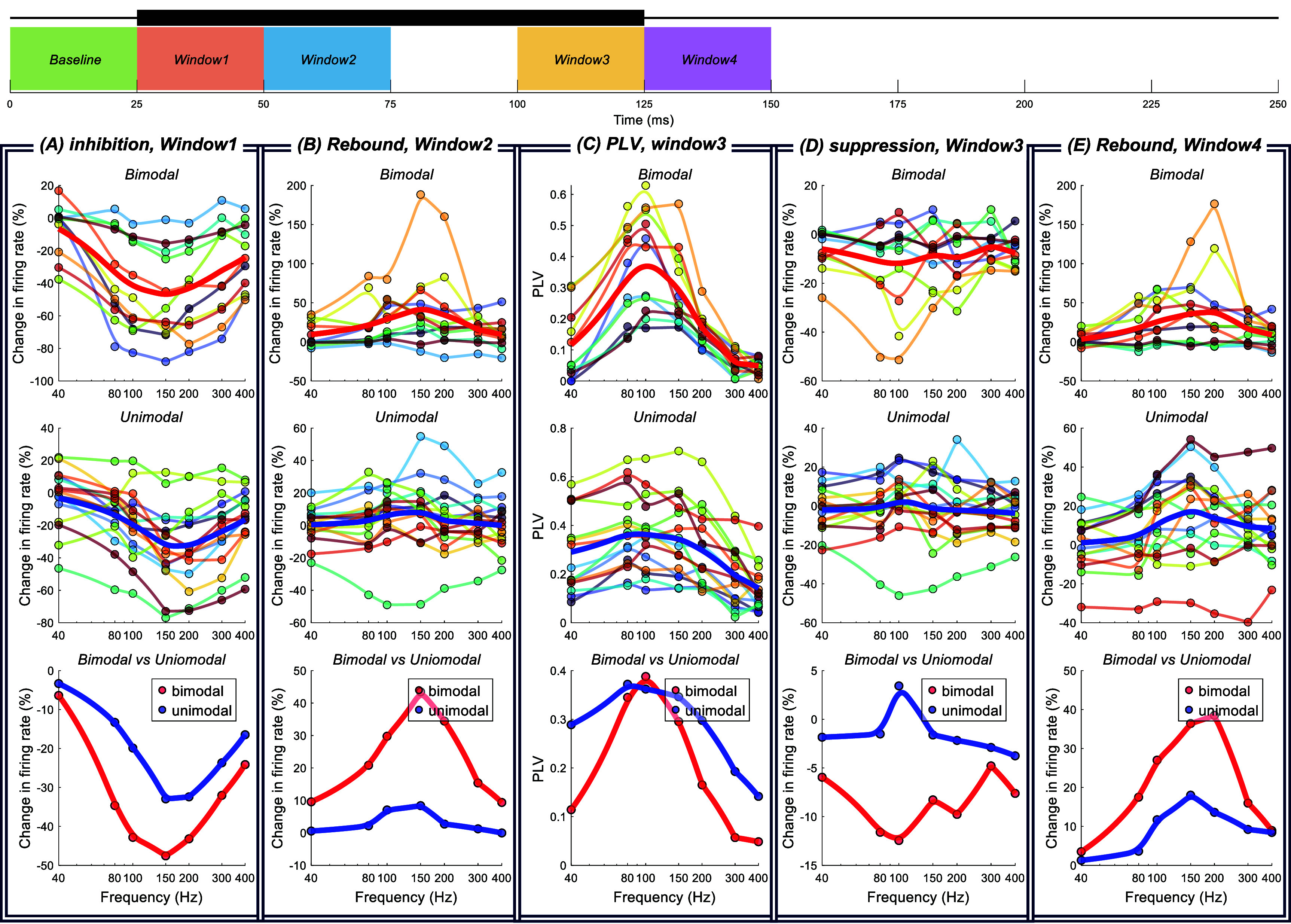
Quantifying different dynamics in TACBS of the CN cells. In all figures, burst frequency ranges from 40 Hz to 400 Hz. A total of 26 cells from 9 rats. In each column the top row shows the result for bimodal CN cells (11 cells from 5 rats) and middle row is the unimodal CN cells (15 cells from 7 rats). The bottom figure shows the comparison of the averages of bimodal and unimodal result. (A): Initial inhibition. (B): Rebound after the initial inhibition. (C): Entrainment during the last quarter of the burst window. (D): Sustained suppression during the last quarter of the burst window. (E): Rebound at the end of the burst window. The definition of the windows with respect to the stimulation cycle are shown with a diagram above the plots. Thick black bar is the AC-burst window.

The peak in the first AC cycle tends to be larger than the others in the PC histogram (figure 11(B)). Correspondingly, the CN cycle begins with inhibition and follows with different levels of entrainment depending on the burst frequency (figure 11(A)). Interestingly, the initial inhibition strength seems to depend on the slope of the rising edge of the stimulus waveform and can be produced even with a single half-cycle sinusoidal (supplementary figure 2). To quantify the inhibition, we calculated the percent change in the firing rate during the first 25 ms of the burst window (*window1* in figure 12(A)) and compared to baseline firing rate sampled from the last 25 ms of data before the burst window (*baseline* in figure 12). Figure 12(A) shows the initial inhibition for bimodal and unimodal CN cells at different burst frequencies. As the frequency is increasing, the inhibition becomes more intense until around 150 Hz where it reaches a maximum and after this point we see a slight weakening. Inhibition is stronger in bimodal CN cells across all frequencies.

##### 3.3.2.2. Sustained suppression and entrainment

Once the initial transient response is over, the rest of the burst window can be considered as the steady-state response to high-frequency AC stimulation. To this end, we looked at the suppression and entrainment (PLV) in the last quarter (last 25 ms) of the burst window (*window3* in figure 12(C)). For calculating PLV we rounded up the window length to contain a whole number of AC cycles. There is only a mild sustained suppression in the bimodal cells (peaking at 100 Hz), and no suppression in the unimodal ones (figure 12(d)).

Despite the overall similarity in the PLV plots for the unimodal and bimodal CN cells, there are interesting and revealing differences. The PLV declines faster in bimodal cells with frequency than the unimodal cells (figure 12(C)). These findings can also be observed in the histogram envelopes in figure 11(A) and can be explained by projections of upward and downward PCs to the CN cells. In unimodal CN cells, only upward or downward PCs putatively project to the CN cell, thus the CN cell should perceive the stimulation with the same frequency as the projecting PCs. In bimodal CN cells, both upward and downward PCs putatively project to the CN cell, thus the CN cell’s activity should decrease both in the rising phase of AC stimulation (because of upward PCs) and the falling phase (because of the downward PCs). As a result, bimodal CN cells are modulated at twice the frequency that the unimodal CN cells are. This is probably one of the reasons behind the faster decline in the entrainment curve of the bimodal CN cells and lower entrainment at higher frequencies.

The strong inhibition at the start of the burst window both in unimodal and bimodal cells (figure 12(A)) and the mild suppression during the burst window in bimodal cells (figure 12(d)) can be leveraged to produce low-frequency modulation in the CN cells, which otherwise requires very large amplitudes to induce using low-frequency sinusoidal stimulation.

##### 3.3.2.3. Rebounds after initial inhibition and at the end of the burst window

A close inspection of figure 11 A reveals that CN firing rates present rebounds at two points; one after the initial inhibition (first rebound) and the other immediately after the burst window (second rebound). To quantify these two rebounds, we compared the firing rate in the burst window within 50–75 ms (*window2* in figure 12(B)) to the baseline firing rate for the first rebound, and the firing rate for 25 ms after the burst window (*window4* in figure 12(e)) to the baseline firing rate for the second rebound. Figures 12(B) and (E) show these two instances of rebounds in the CN firing rates for bimodal and unimodal CN cells as a function of frequency. For both types of cells, the trend is similar where both the first and the second rebounds are stronger in the mid-frequencies and peaks at 150 Hz or 200 Hz. But both rebounds are much stronger with the bimodal CN cells than they are with unimodal CN cells. The fact that the initial suppression was stronger with bimodal cells may be the reason for the first rebound also being stronger in these cells.

### 3.4. Comparison of PC and CN tuning curves

**Figure 13.**
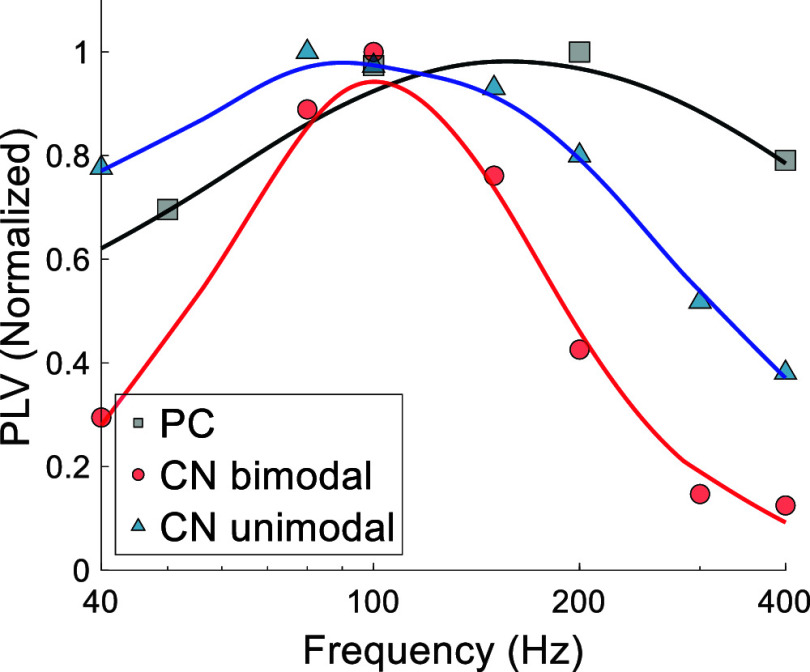
PLV plots for the PCs and the CN cells from 40 Hz to 400 Hz. PLV data from figures 12(C) and 5 are normalized for comparison. Unimodal and bimodal CN cells are plotted separately.

It was previously reported that CN cells could be modulated transsynaptically [13] by entraining the projecting PCs of the cerebellar cortex [12, 18]. In general, the frequency response in the CN cells is similar to the PCs in that the strongest modulation occurs in mid-frequencies. However, the CN cell modulation quickly declines below 50 Hz [13] where PCs do still have some level of modulation (figure 5). More strikingly, the PCs continue to be modulated at frequencies above a few hundred Hertz while the CN modulation plots, for both unimodal and bimodal types, decline faster with frequency (figure 13).

### 3.5. Exploring the effects of different waveforms on CN cell activity

#### 3.5.1. Adding a theta frequency sinusoidal baseline to the high frequency bursts

**Figure 14.**
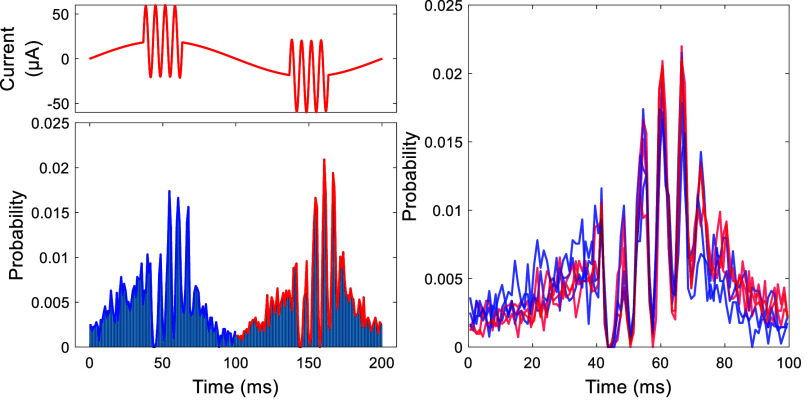
Response of a CN cell to the stimulation with a sinusoidal baseline in the theta band. Left-top: stimulation pattern with 150 Hz sinusoidal superimposed in both positive and negative cycles of a 5 Hz sinusoidal waveform. Left-bottom: spike histograms of a CN cell plotted for a stimulation amplitude of 20 *µ*A, peak, for both sinusoidal components of the waveform. Solid lines are fitted to the bar plots. Right: histograms for the positive (blue) and negative (red) cycles of the theta waveform, merged together for three different theta stimulation amplitudes (0, 10 and 20 *µ*A). Stimulation frequencies (150 Hz) and amplitudes (20 *µ*A, peak) are the same in all three cases.

Because of the clinical relevance of theta-gamma stimulation [14–16], we also questioned if replacing the flat baseline in the TACBS waveform with a sinusoidal would induce a difference in the entrainment pattern by the high-frequency component. A few cycles of high-frequency burst were superpositioned in each half cycle of a 5 Hz sinusoidal with the same amplitude (20 *µ*A, figure 14). There was no discernible difference in modulation between the positive and negative cycles of the theta frequency (figure 14). The results from 11 cells in 4 rats did not show any significant difference between PLVs for the positive and negative cycles at 80 Hz (*p* = 0.30) and 150 Hz (*p* = 0.97) burst frequency (paired t-test). The lack of modulatory effects by the theta frequency sinusoidal baseline agrees with low modulation levels seen at low frequencies in the CN cells [13]. In addition, this result suggests that there are no non-linear effects that would emerge as a result of interaction between the responses to each sinusoidal waveform (i.e. interference). Although larger amplitudes of the baseline sinusoidal could probably yield a difference in high-frequency modulation levels between the two half cycles, this would be impractical because it would saturate the firing rates induced by the high-frequency component.

#### 3.5.2. AM stimulation waveform can eliminate the transient response and induce low-frequency modulation

**Figure 15.**
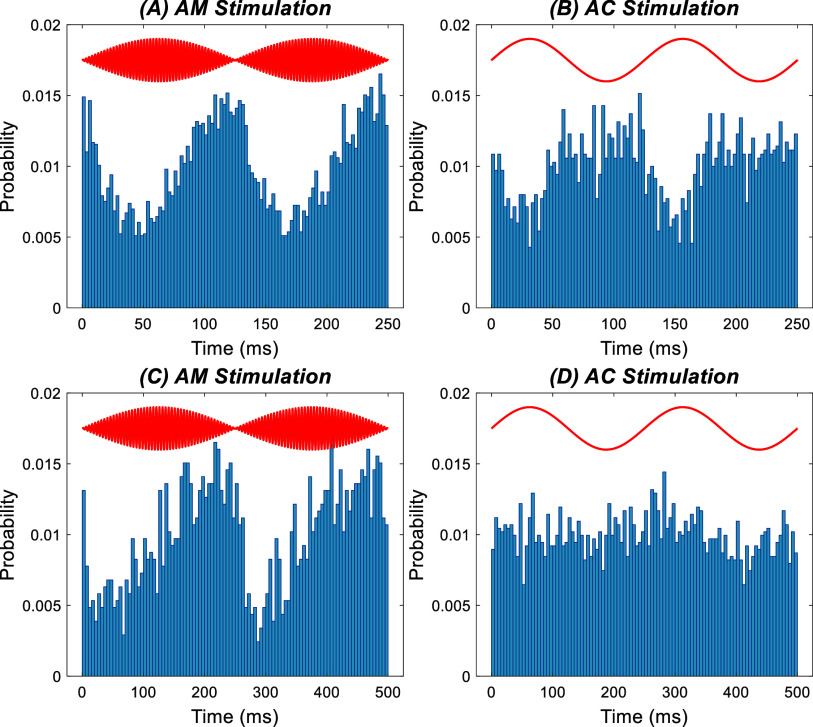
Examples of AM stimulation and comparisons to pure sinusoidal stimulation in two cells (cell 1: (A) and (B) and cell 2: (C) and (D)) from two different rats. (A): 8 Hz AM stimulation with 400 Hz carrier signal, and (B) 8 Hz pure sinusoidal stimulation in the same cell. (C): 4 Hz AM stimulation with 200 Hz carrier signal in a second cell, and (D) 4 Hz pure sinusoidal stimulation in the same cell.

The transient effects with TACBS may be undesirable in some clinical applications. The strong transient effects at the onset and offset of the burst window can be smoothed out by multiplying the stimulation waveform with a theta frequency sinusoid [14, 19, 20]. This amplitude modulated (AM) stimulation waveform was tested to demonstrate that a stronger low frequency modulation can be induced in a CN cell compared to a pure sinusoidal at the same theta frequency (figure 15). The inhibition is produced by the high-frequency component of the stimulation but the overall modulation pattern follows the theta frequency envelope of the AM signal. The results from 6 cells in 2 rats showed a significant difference (*p* = 0.018) between PLV (0.21 ± 0.17) of AM stimulation with carrier frequency of 200 Hz and the PLV (0.13 ± 0.11) of pure sinusoidal stimulations at 4, 8, and 16 Hz (paired *t*-test).

### 3.6. PC responses can be explained by a simple cell model with intrinsic oscillations

The transient response to pulse stimulation was much greater than the steady-state response during the flat portion of the pulse in the PCs. Surprisingly, a simple cell model can explain the exaggerated transient response to a current pulse. In this model, we assumed that the transmembrane potential increases spontaneously as a ramp until it reaches a threshold, at which time a spike is generated, and the transmembrane potential is reset to baseline level to restart the cycle. In this oscillatory model, the slope of the voltage ramp, and thus the ISI, is made to vary stochastically according to the Burr distribution (*c* = 3.5, *k* = 0.5 and α sets the mean firing frequency, MATLAB function), which best fitted the probabilistic distribution of the ISI in our neural data. (The specific probability distribution used is not critical as the Poisson distribution (*λ* = 40), which is commonly used to model neuronal spike trains, produces similar results, data not shown). Then, the subthreshold stimulation signal is added onto the intrinsic membrane oscillations to modulate the firing pattern of the spikes.

**Figure 16.**
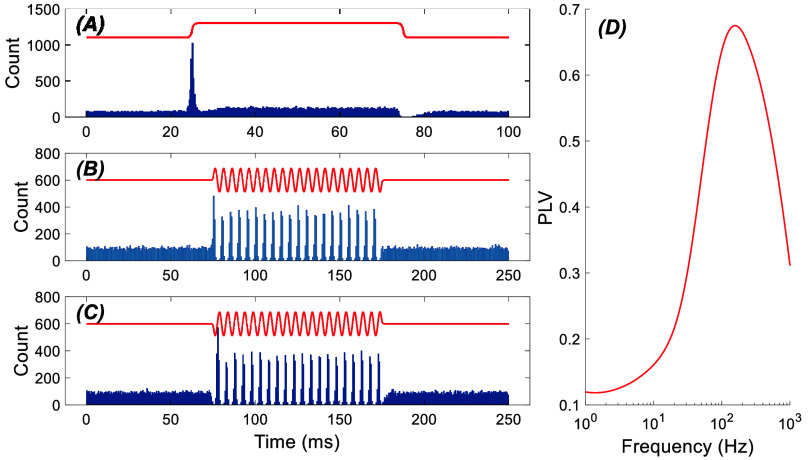
Spontaneously firing cell model (40 Hz), simulating the PC response to different forms of stimulation. (A): 10 Hz pulse stimulation. A low-pass filter with a rise time of 1 ms was applied to the stimulus waveform to simulate the filtering effect of the membrane capacitance. (B): TACBS, 4 Hz theta and 200 Hz gamma frequency. (C): stimulation is reversed to simulate the downward cells. (D): PLV calculated for AC stimulation of the cell from 1 Hz to 1 kHz. In all cases the stimulation amplitude is 20% of the variation of the membrane potential.

This model produced a very similar transient and steady-state responses as well as the AC entrainment profiles that we observed in our PC data (figure 16). A statistical consideration could explain the results. If the stimulation induces a constant voltage change (DC) with an amplitude equivalent to, for instance, 10% of the peak-to-peak membrane potential oscillations, the average firing rate should change about 10%, assuming linearity. But, an instantaneous change in the stimulation (current step) by the same amount can cause the upcoming spikes to occur 10% earlier than expected, increasing the probability of a spike occurring precisely at the moment of transition. For this to statistically be significant, the stimulus needs to be applied a sufficient number of times per unit time (figure 16(A)). This sudden change in spike numbers, though it is a small percentage, statistically constitute a large peak in the histogram plot around the transition time. Then, the instantaneous firing rate goes back to a new baseline (because of steady state response) with a time course that is determined by the mean spontaneous firing rate of the cell. That is, after firing a spike at the rising edge, the next spike will occur with a delay determined by the mean firing rate of the cell (figure 16(A)). At the inhibitory edge of the current step, the exact opposite happens, and the instantaneous drop of transmembrane voltage will prevent the cell from firing in 10% of imminent spikes, which will show up as a strong transient inhibition in the histogram (falling edge in figure 16(A)), etc. The simulation results to TACBS are also similar to the real PC (figures 16(B) and (C)) in that there is a strong entrainment with the largest peak of the histogram occurring at the first cycle.

This simple statistical analysis can also explain why the transient effect gets weaker with slower slopes at the current pulse transitions. As the transition time is increased relative to the cell’s mean ISI, the spike numbers affected by the stimulus edge statistically becomes lower per each unit of time, and the transient response is spread over a longer duration with a smaller peak.

The sinusoidal AC stimulation waveform also consists of a train of rising and falling transitions. Following the same statistical model, the instantaneous changes in the firing rates during the AC cycle is expected to become weaker with decreasing frequencies where the slope in the sinusoidal waveform is becoming less steep. This will result in lower modulation levels at low frequencies (figure 16(d)). At the higher end of the spectrum, the low-pass filter (1 ms rise time) incorporated into the model to simulate the filtering effect of the membrane capacitance forces the PLV to decline with frequency. Overall, a simple stochastic model combined with a linear filter could mimic both the transient and frequency characteristics of the PCs at a basic level.

## 4. discussion

In this paper, we investigated the temporal and frequency dependent modulation characteristics of the cerebellar PCs and CN cells. Transient and steady-state responses to rectangular stimulation waveforms, in addition to computer simulations, provided insight into modulation mechanisms that may be different for high and low frequency AC stimulation, as well as for DC stimulation. Pushing the upper bound of the stimulation frequency (up to 1000 Hz) allowed us to look into the mechanisms underlying the tuning curve in transsynaptic CN modulation reported earlier [13]. We discuss the practical implications of these results for clinical applications of ctACS at the end of the discussion.

### 4.1. Mechanisms of PC modulation

Extracellular e-fields can hyperpolarize or depolarize the cell membrane at the soma [21–23] and alter the firing rate of the cell. The alterations in the transmembrane potential in the PC dendrites could also modulate the post-synaptic currents [22, 24] by the incoming parallel fiber inputs on the PCs (and potentially the MLI) by altering the driving voltage for the post-synaptic currents. The post-synaptic currents then propagate down to the soma electrotonically and alter the firing rate. Furthermore, the e-fields could facilitate synaptic efficacy by hyperpolarizing the pre-synaptic axon terminals [23]. Computer simulations also predicted that the synaptic strengths, and thus the post-synaptic spikes induced by parallel fiber inputs, play a substantial role in modulation of instantaneous firing rates by DC e-fields [21]. In this study, we are unable to separate the direct effects of the e-field on the PC firing rates and the effects on the synaptic inputs that would indirectly alter the PC firing rates. The parallel fiber-to-PC synapses, which is AMPA mediated, should be mostly unaffected by the ketamine anesthesia, an NMDA receptor antagonist. Future experiments can investigate the relative contributions of the direct and synaptic modulatory effects by chemically blocking the parallel fiber and MLI inputs.

The PC cells could be entrained at frequencies below 5 Hz, though with currents larger than those used in this study [18]. As we now expand the frequency range, we see that it takes much less current to modulate PCs at high frequencies. In addition to the mechanisms explained in the results section based on simple statistics in an oscillatory cell model, the PC modulation may also be actively suppressed at low frequencies of AC stimulation and for DC stimulation. The inhibition from the MLI can potentially dampen the modulatory effects of the low frequency or constant e-fields on the PCs. The MLI with dendrites extending along the direction of the e-field were modulated at low frequencies almost as strongly as the PCs in an *in vitro* turtle preparation [25]. The type of MLI in the cited study might be the basket cells since depletion of the basket cells in knockout mice alters the PC-SS firing rate while the depletion of stellate cells only makes the PC-SS firing pattern more regular [26].

The strong transient response vs. small steady-state response could also result from excitation of the MLI along with the PC by the stimulation current. Inhibition by MLI would counterbalance the excitation of the PC and suppress the change in the average firing rate. Depending on how quickly MLI output can change at the onset of the current step, there may be a transient increase in the PC firing before MLI inhibition kicks in. Normally, this delayed PC inhibition by MLI serves to sharpen the PC response spatiotemporally to parallel fiber inputs [26, 27]. It is not clear at this point how much, if any, the MLI stimulation may be contributing to the observed low-frequency and steady-state responses in PCs.

### 4.2. AC and DC stimulation may be operating through different mechanisms

Computer simulations suggested that the DC e-fields would alter the silence period following the PC-CS in a polarity specific manner [21]. The lack of modulation in the SS silence following the CSs in our data is probably because the AC stimulation does not bias the average PC resting membrane voltage over time, but induces only temporary changes in synch with the AC cycle. Indeed, direct currents and very low frequency stimulation may be operating through a different mechanism than the high-frequency AC stimulation, according to our simulation results (figure 16). A constant voltage bias could slowly shift the mean firing rate of the cell over a time frame that is much longer than a single cycle of high frequency stimulation.

This slow modulation mechanism in the mean firing rate of the PC may be an emergent property of the neural circuitry with recurrent pathways that provide positive feedback to the PC by one of several feedback loops between other brain areas and the cerebellar cortex. Another possibility is an intrinsic mechanism within the PC that leads to a slow shift in the mean firing rate. tDCS was predicted to alter the voltage-gated sodium channel dynamics and thereby the bursting pattern of the PCs [21]. It is not clear from our data whether extrinsic or intrinsic mechanisms are causing the elevated modulation at frequencies below 5 Hz that we observed in a few cells (figure 5(B)).

The asymmetric modulatory effects of anodic vs. cathodic stimulation were predicted for tDCS [21]. Jumping of the spike initiation site from the soma to the depolarized dendrites under cathodic stimulation was proposed as the potential mechanism of this asymmetry [22]. We did not observe asymmetric modulation with sinusoidal waveforms when the stimulation amplitudes were small enough not to cause saturation of the firing rates. The rectangular stimulation produced asymmetric transient PC and CN responses primarily because the firing rates were driven to extremely low or high levels at the falling and rising edges of the stimulation. This probably highlights another mechanistic difference in DC and AC modulation. AC stimulation only modulates the timing of the spikes by synchronizing them to the stimulation cycle. The synaptic inputs and the intrinsic factors continue to dominate the baseline firing rate of the cell. DC stimulation, on the other hand, primarily affects the baseline firing rate, and potentially the bursting pattern and regularity of the spikes [21] without dictating their exact timing. Therefore, the asymmetric modulation by cathodic and anodic stimulation may be a feature of DC stimulation that is related to the mean firing rate of the cell.

### 4.3. PCs entrain in 180° phase difference

The net effect of the e-field depends on the orientation of the somatodendritic axis of the cell relative to the field and the cellular morphology, particularly the pattern of the dendritic arborization [28, 29]. Anodic currents from surface electrodes hyperpolarize the apical dendrites near the surface and depolarize the basal dendrites and the soma, and cathodic currents do the opposite [21, 23]. Cells whose somatodendritic axis is longer in the direction of the e-field experience larger membrane polarization at the soma and their spiking is entrained stronger in AC fields [28, 30].

With the extensive dendritic arborization of the cerebellar PCs in the parasagittal plane, the net polarization effect can vary depending on the relative position of the cell and the orientation of its dendritic branches with respect to the stimulation electrode. Moreover, the e-field with surface monopolar electrodes can have large tangential components parallel to the cortical surface [23]. Our group reported that e-fields in the rostro-caudal direction are more effective for PC modulation than the other two orthogonal directions [18]. However, quite interestingly, the direction of the modulation could be opposite in PCs found in close proximity to each other. This observation is confirmed with the current data that even PCs found near the same stimulation electrode could be modulated in opposite directions. It is difficult to explain this result by differences in the pattern of the PC dendritic arborizations and orientation of the dendritic branches in the e-field. A more likely explanation is provided by the surround inhibition mechanism through the PC axon collaterals in the same parasagittal zone [31]. The PC collaterals can directly inhibit other PCs (or indirectly disinhibit them by inhibiting MLIs). If the indirect transsynaptic effects on a PC via these collaterals is stronger than the direct excitatory effect by the e-field, the PC-SS activity may be modulated in opposite directions to other PCs that are found in the same parasagittal zone in close proximity. A slight difference in favor of direct or indirect effect could also be exaggerated through reciprocal connections between those PCs [31].

### 4.4. CN modulation

#### 4.4.1. Transient responses

The initial inhibition in the CN with rectangular pulses or theta-burst stimulation and the fast recovery from this initial response follows the pattern of the transient PC response, considering that it is a GABAergic connection (figure 11). A synaptic accommodation mechanism has also been reported that the size of post-synaptic currents in the CN cell decreases by train stimulation of PC axons [32]. But this accommodation process takes place in much longer time frames, even with high frequency (100 Hz) train stimulation, than those we observed in recovery of the initial inhibition. Moreover, stimulation with only a half sinusoidal (supplementary figure 2) induces the same initial inhibition pattern in the CN cell as the TACBS with multiple cycles. This indicates that the first rising edge of the AC stimulation waveform is responsible for the initial CN inhibition being stronger than the following ones. These mechanisms in addition to the rebound mechanisms reported in the CN [33–36] may be giving rise to the complex dynamics of CN response to theta-burst stimulation with multiple phases of inhibition and rebound.

#### 4.4.2. Frequency response

The CN cells have lower modulation at the low and high ends of the frequency range [13] than the PCs, as shown by comparison of their PLV-frequency plots (figure 13). The lower CN modulation at low frequencies can be a combination of several factors: (1) low levels of modulation in PCs at low frequencies in turn leads to low modulation levels in the CN cells, (2) accomodation to the PC inhibition [32] makes the CN cells desensitize to slow changing firing rates in the PCs, (3) the AC cycle is too long at low frequencies in order to achieve sufficient PC synchrony and spike coincidence for post-synaptic entrainment of the CN cells [37]. A feedforward inhibition mechanism was also proposed as an explanation for the intrinsic gamma oscillations (30–90 Hz) being more effective than lower frequencies for the entrainment of post-synaptic networks [38]. Specifically, an inhibition cycle begins with a few milliseconds delay in the post-synaptic circuits following an excitatory phase. The duration of this excitatory-inhibitory cycle is such that it allows the post-synaptic cells to be entrained selectively by inputs that are bursting in gamma frequencies [39].

On the other hand, the mechanism for low CN modulation levels at high frequencies can be rationalized as follows: At frequencies above the PC baseline firing rates, the PCs start skipping the AC stimulation cycles and different subgroups of PCs projecting to the same CN cell can fire in different cycles of the signal (and at subharmonic frequencies). Because the SS from different PC subgroups are now spread over time more uniformly, CN entrainment turns into suppression at high frequencies. The intrinsic jitter due to the difference in the arrival times of the spikes from projecting PCs will make the spike distribution even more uniform. This agrees with the reports showing that a high rate of firing in the PC inputs induces tonic depression in the PC-CN synapse [32].

### 4.5. Translational implications

#### 4.5.1. Stimulation waveform for transsynaptic CN modulation

The e-fields with surface electrodes decrease exponentially by depth and thus direct modulation of the CN cells may not be feasible with non-invasive methods [21] without generating extreme e-fields in the cerebellar cortex. Transsynaptic modulation of the CN overcomes this problem [13] by targeting the PCs of the cerebellar cortex rather than the cells of the nuclei that are deeper in the cerebellum, and may present an alternative to CN stimulation with implantable deep brain stimulation electrodes [40–42]. Transsynaptic CN modulation at theta frequencies is possible with TACBS pattern, despite the fact that continuous sinusoidal signals at theta frequencies are not efficient to entrain the PCs or the CN cells. This can explain the positive results reported with theta-gamma stimulation in human trials [14–16].

The functional implications of the transient inhibition and rebound events at the onset and offset of the theta cycle are not clear. If the modulatory effects of these transient responses at the cerebral sites projected by the CN outputs are not desired, AM stimulation or other waveforms with smoothed edges [19] that can eliminate the transient effects may be preferred in clinical trials. Depending on the choice of the high frequency component of the waveform, the CN cells can be entrained or suppressed, which may have different functional implications also at the projecting sites. CN suppression at theta repetition rates may be desired to modulate CBI in certain cerebello-cerebral pathways, whereas entrainment of the CN cells, which in turn can entrain the projecting cerebral sites, may be needed in others. The aberrant activities that characterize various neurological disorders that implicate the cerebellum may be corrected using either one of these methods depending on the nature of the signals. Suppression of aberrant activity may be used in epilepsy or essential tremor patients, while entrainment of the spontaneous neural firing may work more effectivity in other forms of motor disorders, such as ataxia, dysmetria or dystonia.

The much lower DC response relative to high-frequency AC stimulation, both in PCs and CN cells, may look surprising at first sight considering the sizeable number of reports on the clinical effects of cerebellar tDCS (see [43] for a review). In this study, we only investigated immediate effects of DC stimulation using short duration direct currents, i.e. rectangular pulses. Our results do not contradict the reported tDCS effects, which are typically applied over minutes and longer. It is interesting to note, however, that the current amplitudes required for PC and CN cell spike entrainment with AC stimulation are much lower than the current amplitudes needed for alteration of the average spike rates with DC. The AC stimulation may not have any cumulative and/or post-stimulus effects [10] since it does not alter the average firing rates, whereas the DC stimulation can produce changes in the mean firing rate of the cells over time and the cumulative and post-stimulus effects may occur. On the other hand, the AC stimulation may be more suitable for modulation of brain oscillations and improving information transfer between different brain sites by episodic synchronization of spontaneous activity [39, 44].

#### 4.5.2. Spikes locking to subharmonics

The PC cells lock to subharmonics of the stimulation frequency by skipping cycles as the stimulation frequency is increased above the spontaneous rate of firing. This skipping mechanism can divide the cells into subpopulations depending on the specific cycles that they fire and the ones they skip. A group of cells, for instance, may fire in the odd cycles while another group may choose the even ones. There may potentially be multiple groups of cells depending on how many folds the stimulation frequency is raised above the spontaneous rate. High frequency stimulation may be leveraged to synchronize groups of cells at interleaving time points in order to keep the global synchrony under control amongst the PC cells, which may, for instance, lead to tremor in muscle recruitment. In fact, interleaved synchrony amongst the motor neurons is one of the proposed intrinsic mechanisms for producing smooth motor movements through the cerebello-cerebral pathway without causing tremor [45].

### 4.6. E-field strengths

The modulation thresholds were found to be around 15–20 mV mm^−1^ both for PCs and MLI where a uniform e-field was applied across the cerebellar cortex *in vitro* by Chan and Nicholson [25]. In our case, the estimated e-field at a depth corresponding to the PC soma (∼300 *µ*m) for a 10 *μ*A current delivered through a monopolar surface electrode (0.25 mm diameter) would be about 36 mV mm^−1^ assuming homogeneous cerebellar conductivity of 0.42 S m^−1^ [46] according to volume-conductor equations [47]. This e-field strength, which is about twice the threshold range measured by Chan *et al*, is predicted by this theoretical model to decline to 0.19 mV mm^−1^ at the depth of CN (∼4.5 mm). In our data, we occasionally found PCs modulating at even less than 10 *μ*A, but in general the thresholds were higher than 20 *μ*A. However, we refrain from presenting qualitative threshold analysis because the exact strengths of the e-fields at the location of the PCs were not measured. Overall, these predicted e-field values suggest that the modulation of the CN cells reported here should primarily be a transsynaptic effect through the PC axons, and not a direct effect of the e-field.

## 5. Conclusion

The PC-SS can be modulated at frequencies as high as 1000 Hz by skipping some of the AC stimulation cycles. The direction of modulation in PCs may be 180° different (upward and downward PCs), even in nearby cells. The combination of upward and downward PCs give rise to bimodal distribution in the CN cell spike histograms locked to the AC stimulus cycle and thereby they are modulated at twice the frequency of PC modulation. The CN entrainment that is seen at mid-/frequencies turns into sustained suppression at high frequencies of AC stimulation. This phenomenon can be leveraged to modulate the CN cells at the theta frequencies using the TACBS waveform in order to overcome the poor modulation with low-frequency sinusoidal waveforms. Strong transient responses occur in the CN firing rates at the onset and offset of the stimulation, both with TACBS and rectangular pulse stimulation. Comparative analysis of pulse and AC responses, in light of computer simulations, suggests that AC and DC stimulation at subthreshold level may operate through different mechanisms. Overall, these results may guide clinical trials of ctACS for the choice of the stimulation frequency and help design alternative waveforms by offering insight into temporal and frequency domain characteristics of cerebellar neuromodulation at the single cell level. These transient and steady-state characteristics can further be utilized to produce the desired modulatory effects at the cerebral sites projected by the cerebellar outputs.

## Data Availability

All data that support the findings of this study are included within the article (and any supplementary files).
